# Frequency and Age-Related Changes in Corneal Astigmatism in Cataract Surgery Candidates at a Training Hospital in Turkey

**DOI:** 10.3390/medicina62010231

**Published:** 2026-01-22

**Authors:** Alper Can Yilmaz, Bagim Aycin Cakir Ince, Onder Ayyildiz, Fatih Mehmet Mutlu

**Affiliations:** 1 Department of Ophthalmology, Gulhane School of Medicine, University of Health Sciences, 06010 Ankara, Turkey; dronderayyildiz@gmail.com (O.A.); fmmutlu@hotmail.com (F.M.M.); 2 Department of Ophthalmology, Ufuk University, Ridvan Ege Hospital, 06520 Ankara, Turkey; dr.aycin@hotmail.com

**Keywords:** astigmatism, cataract surgery, corneal astigmatism in Turkish population, keratometry, toric intraocular lens

## Abstract

*Background and Objectives*: To evaluate the magnitude, axis and age-related changes in corneal astigmatism in patients before cataract surgery. *Materials and Methods*: In this retrospective, cross-sectional, and observational study, data from 2152 eyes that underwent phacoemulsification were evaluated. Keratometric values were obtained using the IOL Master 500 device. The frequency, magnitude and axis of corneal astigmatism were determined. The astigmatism axis was categorized as with the rule (WTR), against the rule (ATR), and oblique astigmatism. Quantitative analysis was performed using the power vector method (J0 and J45). The distribution and characteristics of corneal astigmatism data according to age were analyzed. *Results*: The mean age of the patients was 70.56 ± 8.88 years (range 40–94 years) and 1010 (46.9%) were males. Mean corneal astigmatism, J0 and J45 values were 0.96 ± 0.72, 0.05 ± 0.51, 0.01 ± 0.30 diopters (D), respectively. The most common range of magnitudes was 0.50–0.99 D with 38.8%, followed by <0.50 D (25.3%), 1.00–1.49 D (20.3%), and 1.50–1.99 D (8.7%). The cubic regression curve showed a U-shaped nonlinear relationship between age and corneal astigmatism (*p* < 0.001). The most common type of astigmatism was WTR with 43.4%, followed by ATR with 37.5% and oblique astigmatism with 19.1%. With the increase in age, the astigmatism axis gradually changed from WTR to ATR. There was a linear trend in the rate of these types of astigmatism across age groups (*p* < 0.05). Additionally, in patients under 65 years of age, WTR astigmatism was negatively correlated with age, while in patients 65 years of age and older, ATR astigmatism was positively correlated with age (r = −0.217, *p* < 0.001; r = 0.153, *p* < 0.001, respectively). Linear regression analyses revealed that the J0 value decreased significantly with age, whereas J45 showed no significant relationship. Specifically, J0 decreased by 0.014 D per year of age (95% confidence interval [CI], 0.011–0.016; *p* < 0.001). *Conclusions*: The results obtained in this study may provide information to guide surgeons in the management of astigmatism and the choice of toric intraocular lens in cataract surgery.

## 1. Introduction

Refraction examination is important before cataract surgery. Today, considering modern surgical techniques and developing intraocular lens (IOL) technologies, the term ‘Refractive cataract surgery’ has come to the fore [1]. In this regard, the measurement of corneal astigmatism before surgery and its management during surgery have a wide place in the practice of cataract surgeons [2]. Previous studies have reported that a substantial proportion of cataract patients present with clinically significant corneal astigmatism, with rates of anterior corneal astigmatism exceeding 1.0 diopter (D), 1.5 D, and 2.0 D ranging from 32.5–45.5%, 21.0–26.2%, and 8%, respectively [3,4,5,6,7]. Limbal relaxing incisions (LRI), opposite clear corneal incisions (OCCI), or toric IOLs are used to correct corneal astigmatism [8,9,10].

Factors such as the magnitude of the astigmatism and its axis determine which of the astigmatism corrective methods will be preferred [8,9,10]. For this reason, it is important to make accurate preoperative measurements. Nowadays, optical biometry devices based on the principle of partial coherence interferometry and optical low coherence reflectometry are widely used [11]. It has been proven that devices using these technologies make more accurate and sensitive measurements than traditional methods [12,13].

Although several population-based studies have reported the prevalence and axis distribution of corneal astigmatism, data directly comparing age-related astigmatic patterns across different geographic regions remain limited [14,15]. The aim of the present study is to evaluate the frequency, magnitude, type, and age-related distribution of corneal astigmatism measured using the IOL Master 500 in a large cohort of cataract surgery candidates. By providing detailed demographic and astigmatism-related data, this study aims to support surgical planning and to estimate the proportion of patients who may benefit from astigmatic correction, particularly toric IOL implantation. In addition, the findings are discussed in the context of previously published data from different countries in order to highlight geographic similarities and differences in corneal astigmatism patterns, with a specific focus on age-related changes.

## 2. Materials and Methods

### 2.1. Study Design

In this retrospective, cross-sectional and observational study, data on 2152 eyes that underwent cataract surgery between July 2023 and April 2024 at Gulhane School of Medicine, Department of Ophthalmology, were evaluated. The study was conducted in accordance with the ethical principles of the Declaration of Helsinki and was approved by the Scientific Research Ethics Committee of the University of Health Sciences (approval number 2025-193).

### 2.2. Examination Procedure and Data Collection

Before the surgery, patients underwent a comprehensive ophthalmologic examination, including corrected distance visual acuity, slit-lamp microscopy of the anterior segment, dilated fundus examination, and intraocular pressure measurement. The IOL Master 500 device (Carl Zeiss Meditec AG, Jena, Germany) was used for keratometric measurements. IOL Master 500 uses integrated autokeratometer to measure keratometry indices [12]. The data in this study, including demographic information, ophthalmological and systemic examination findings, were obtained from patient files and Gulhane Research and Training Hospital’s electronic medical record system. In addition to demographic data such as age and gender in this study, axial length, anterior chamber depth, IOL power, keratometry measurements, magnitude and axis of corneal astigmatism and number of patients implanted with toric IOL were recorded. IOL Master device measurements, including data on corneal astigmatism, were obtained by making at least three automatic measurements by an experienced technician. The average of three consecutive measurements was obtained and used for the analysis. History of intraocular surgery (including refractive surgery), ocular trauma, contact lens wear, history of corneal disease (keratitis, corneal opacity, corneal ectasia, etc.), and any ophthalmic or systemic condition affecting IOL Master measurements were determined as exclusion criteria from the study.

### 2.3. Definitions

All corneal astigmatism values were converted to power vectors (J0 and J45) to quantitatively evaluate the magnitude and axis of astigmatism [16]. This method, which converts corneal astigmatism from a spherocylinder notation to a power vector notation, is known the Fourier transformation [16] and is formulated as follows. J0 = (−C/2) cos2α, J45 = (−C/2) sin2α, where C is the negative cylinder power, α is the cylinder axis, and J is the Jackson astigmatic vectors; J0 refers to cylinder power fixed at the 90° and 180° meridians, and J45 refers to cylinder power fixed at the 45° and 135° meridians [17]. The positive and negative values of J0 represent with the rule (WTR) and against the rule (ATR) astigmatism, respectively; and the positive and negative values of J45 represent clockwise and counter-clockwise oblique astigmatism, respectively [18]. According to these measurements, corneal astigmatism was divided into three groups. When the steeper axis was between 60–120°, it was categorized as WTR, when it was between 0–30°/150–180°, it was categorized as ATR, and the group other than these was categorized as oblique astigmatism [16].

### 2.4. Statistical Analysis

Data analysis was performed with SPSS 27.0 program (IBM Corp., Armonk, NY, USA) and studied with 95% confidence level. The normality of continuous variables was evaluated using the Shapiro–Wilk test. Corneal astigmatism measurements were deemed not to show a normal distribution. Therefore, non-parametric methods were used in the analyses. Frequency (*n*) and percentage (%) were given for categorical (qualitative) variables, mean, standard deviation (SD), minimum, maximum and median statistics were given for numerical (quantitative) variables. In the study, Mann–Whitney U and Kruskal–Wallis tests were used to compare the measurements according to the groups, Chi-square test was used for the relationships between grouped variables, and Spearman’s correlation test was used for the relationship between age and corneal astigmatism. In addition, in order to evaluate the possible non-linear relationship between age and corneal astigmatism, a regression model including cubic polynomial terms of age was created and the trend curve was visualized with 95% confidence interval. Additionally, the power vector method, J0 and J45 values, and linear regression models were used to evaluate the association between age and astigmatism. A *p* value of 0.05 or less was considered statistically significant.

### 2.5. Test–Retest Reliability for Keratometric Measurements

Test–retest reliability for keratometric measurements was assessed using an intra-class correlation coefficient (ICC). ICCs were calculated using a two-way mixed model with absolute agreement, based on the mean of multiple measurements. ICC values ranged from 0 to 1, where low ICC values represent low levels of test–retest reliability, and high ICC values represent high levels of test–retest reliability. Specifically, in the present study, ICC values of <0.50 were considered to represent poor reliability; values of 0.50–0.75 represented moderate reliability; values of 0.75–0.90 indicated good reliability; and values of ≥0.90 represented excellent reliability.

## 3. Results

In this study, data were obtained from 2231 eyes of 2231 patients. Forty-six patients were excluded from the study, including 7 patients with a history of ocular trauma, 16 patients with a history of intraocular surgery, 11 patients with corneal opacity, 2 patients with keratoconus, 4 patients with pterygium extending to the optic axis, and 5 patients with corneal dystrophy, and 1 patient with anterior microphthalmus. In addition, 33 patients had ophthalmic or systemic conditions that prevented IOL Master measurements. As a result, data from 2152 eyes were included in the statistical analysis.

**Figure 1:**
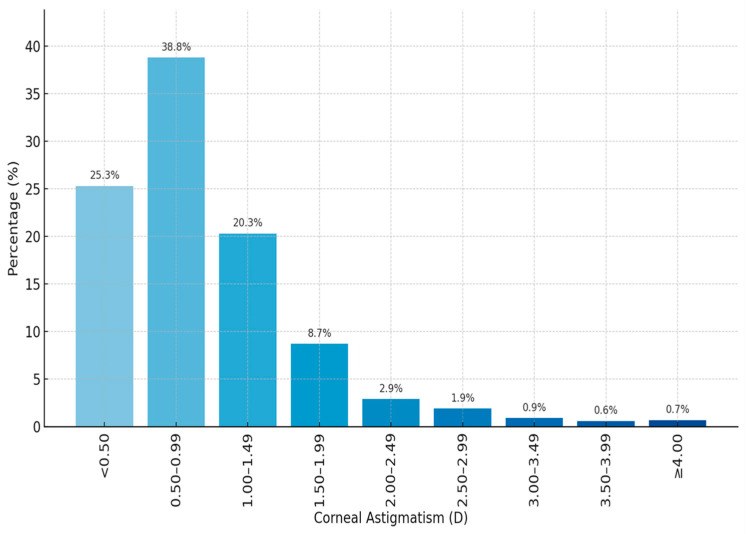
The frequency distribution of corneal astigmatism magnitudes. D, diopter.

The mean age of the patients was 70.56 ± 8.88 years (range 40–94 years) and 1010 (46.9%) were males. Mean corneal astigmatism was 0.96 ± 0.72 D (range 0–8.31 D). The mean flat keratometry (K1) value was 43.32 ± 1.60 D, while the mean steep keratometry (K2) value was 44.28 ± 1.66 D. Test–retest reliability values for keratometric measurements were excellent (ICC = 0.943). Mean J0 and J45 values were, 0.05 ± 0.51 D (range −2.29 to 4.38 D), 0.01 ± 0.30 D (range −1.96 to 1.95 D), respectively. When evaluated in terms of astigmatism axis, the most common type of astigmatism was WTR with 43.4%, followed by ATR with 37.5% and oblique astigmatism with 19.1%. Demographic and clinical characteristics of the study population are shown in Table 1. The frequency distribution of corneal astigmatism magnitudes in the study population is shown in Figure 1. The most common magnitude range was 0.50–0.99 D with 38.8%, followed by <0.50 D (25.3%), 1.00–1.49 D (20.3%), and 1.50–1.99 D (8.7%). The magnitude of corneal astigmatism was <2.00 in 2001 (93.1%) eyes and ≥2.00 D in 151 (6.9%) eyes. The magnitude of corneal astigmatism was 4.00 D or above in only 15 (0.7%) eyes.

**Figure 2:**
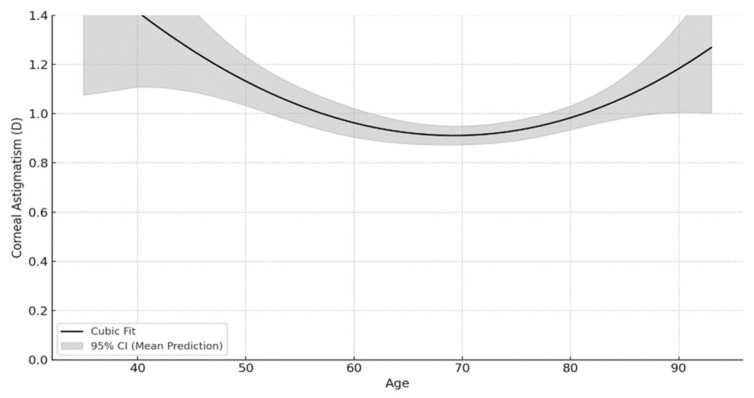
Cubic regression curve showing the association between the magnitude of corneal astigmatism and age. Corneal astigmatism is indicated by a solid line, and 95% confidence interval is indicated by the shaded area. D, diopter; CI, confidence interval.

In the comparison among age groups, the level of corneal astigmatism was highest in the 40–49 age group (*p* < 0.05). The median corneal astigmatism was found to be 1.04 (0.00–3.14) in the 40–49 age group, 0.86 (0.1–4.98) in the 50–59 age group, 0.74 (0.1–5.62) in the 60–69 age group, 0.79 (0.1–8.31) in the 70–79 age group, and 0.89 (0.11–5.02) in the 80 and older age group. According to the post hoc analysis results, the mean corneal astigmatism value of the 60–69 age group was significantly lower than both the 50–59 age group (*p* = 0.002) and the ≥80 age group (*p* = 0.003). The cubic regression curve (Figure 2) created in the study to model the association between age and corneal astigmatism magnitude reveals that this association is not linear (*p* < 0.001). According to the model obtained, corneal astigmatism levels are relatively high at younger ages (especially between the ages of 40–60). As age increases (between the ages of 60–70), a significant decrease in corneal astigmatism is observed. After the age of 70, an increase in astigmatism occurs again. This shows that there is a U-shaped nonlinear association between age and corneal astigmatism.

**Figure 3:**
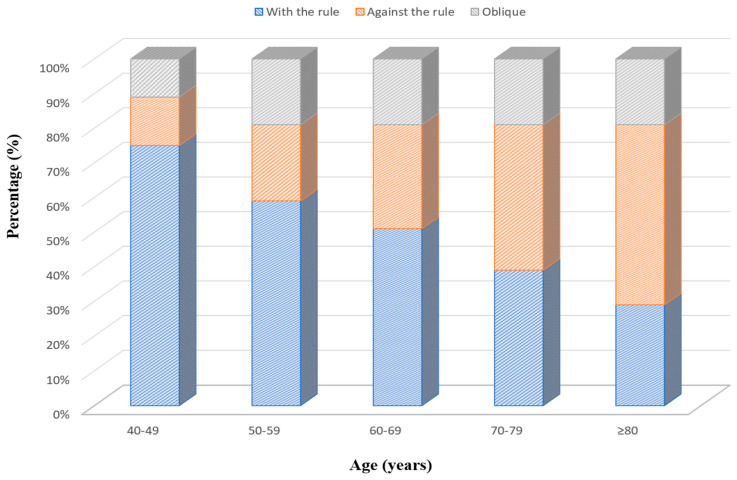
Association between age and corneal astigmatism types.

The distribution of astigmatism types according to age groups is shown in Figure 3. ATR astigmatism increased significantly with age. This type of astigmatism was observed at the lowest rate of 14% in the 40–49 age group and at the highest rate of 52.2% in the ≥80 age group. WTR astigmatism decreased with age. The highest rate was seen in the 40–49 age group with 74.4%, and the lowest rate was seen in the ≥80 age group with 28.7%. There was a linear trend in the rate of these types of astigmatism across age groups (*p* < 0.05). Oblique astigmatism showed a more constant course among age groups, with the highest rate of 19.5% in the 70–79 age group and the lowest rate of 11.6% in the 40–49 age group. According to the post hoc analysis results, the ≥80 age group was significantly different from all other age groups in terms of the rate of astigmatism types (*p* < 0.001). The 70–79 age group was significantly different from all other age groups except the ≥80 age group (*p* < 0.001). ATR astigmatism was observed at a higher rate (41.9%) in patients aged 65 years and over, while it was observed at a lower rate (22.2%) in patients under 65 years of age (*p* < 0.001). WTR astigmatism was observed at a higher rate in patients under 65 years of age (58.3%) and at a lower rate in patients aged 65 years and over (39.1%) (*p* < 0.001). Oblique astigmatism rates were similar in both age groups (19.5% vs. 19.0%), and no significant difference was observed.

There was a positive and significant correlation between age and corneal astigmatism in the ATR group (r = 0.148, *p* < 0.001). No significant association was observed in the WTR and oblique astigmatism groups. In patients aged <65 years, there was a negative, significant correlation between age and corneal astigmatism in the WTR group (r = −0.217, *p* < 0.001). The correlation was not significant in the ATR and oblique astigmatism groups. In patients aged ≥65 years, a positive and significant association was found between age and corneal astigmatism in the ATR group (r = 0.153, *p* < 0.001). The association was not significant in the WTR and oblique astigmatism groups. The correlation analysis between corneal astigmatism and age in three types of astigmatic axis is shown in Table 2.

**Figure 4:**
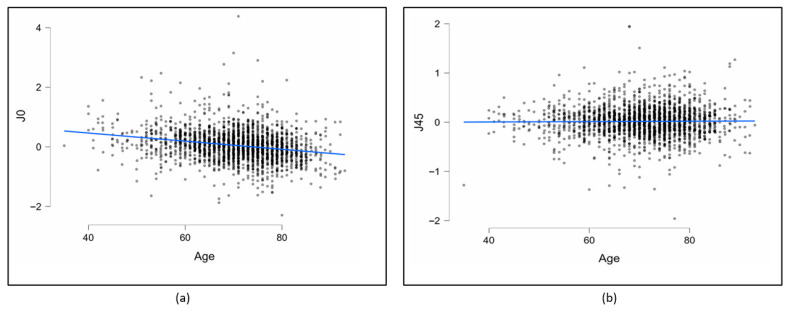
Scatterplots of J0 (**a**) and J45 (**b**) against age in all eyes. (J0: Jackson cross-cylinder, axes at 90° and 180°, J45: Jackson cross-cylinder, axes at 45° and 135°).

Figure 4 shows the scatterplots of J0 and J45, respectively, against age in eyes. Linear regression analyses revealed that the J0 value decreased significantly with age, whereas J45 showed no significant relationship. Specifically, J0 decreased by 0.014 D per year of age (95% confidence interval [CI], 0.011–0.016; *p* < 0.001). Moreover, 5.5% of the variation in J0 could be explained by age.

## 4. Discussion

This study reveals the characteristics and age-related changes in corneal astigmatism in a relatively large series of patients who were candidates for cataract surgery in a teaching hospital in Turkey. It is important because it is one of the limited number of studies reported from Turkey and shares current data from this geography. These findings have direct implications for surgical planning in cataract surgery, as uncorrected residual astigmatism is known to adversely affect postoperative visual outcomes and patient satisfaction [19]. Hasegawa et al. [19], reported that uncorrected distance visual acuity (UDVA) in pseudophakic eyes significantly correlated with the magnitude of residual refractive astigmatism, with UDVA decreasing by 0.193 logMAR for every 1.0 D of cylinder, highlighting the clinical relevance of even low degrees of uncorrected astigmatism. Moreover, the type of residual astigmatism has been shown to affect visual outcomes, with eyes exhibiting with-the-rule astigmatism achieving better postoperative UDVA compared with those with against-the-rule or oblique astigmatism, even after adjusting for age and other confounding factors [19]. These findings emphasize the importance of accurately identifying both the magnitude and axis of preoperative corneal astigmatism [1].

In the present study, 64.1% of patients had corneal astigmatism of less than 1.0 D. Previous reports have shown that small clear corneal incisions may induce flattening from 0.5 up to 0.62 D along the incision axis, suggesting that low degrees of astigmatism may be partially corrected through incision placement [20,21]. Therefore, patients with low astigmatic values may benefit from on-axis clear corneal incisions. However, the effectiveness of this approach may be influenced by several factors, including incision location, surgeon comfort, and operating microscope positioning, which may limit its practical applicability in some cases. The proportion of eyes with ≥1.0 D corneal astigmatism in this study was 36.0%, which is within the range reported in the literature (35.0–67.39%) [22,23,24]. In Turkey, Duman et al. [25] reported a lower rate of 27.9% in a cohort of 1233 eyes, whereas a recent population-based screening study reported a prevalence of 38.0% [26]. This variability may be due to geographic variation, different demographic characteristics, and genetic and environmental factors.

Toric IOLs are safe and effective in the treatment of 1D and above corneal astigmatism and can be used as a first-line treatment option [27,28]. In this study, more than one-third (36.0%) of patients were potential candidates for toric IOL implantation, which is consistent with the rates reported by Wu et al. [14] (38.79%) and Joshi et al. [15] (32.5%). In the only study conducted in Turkey where corneal astigmatism characteristics were analyzed before cataract surgery, Duman et al. [25] reported this rate as 27.9%. In this study, among patients with toric IOL indications, 41.4% actually received a toric IOL. Reporting these real-world implantation rates may provide useful information for surgical planning, institutional resource allocation, and IOL manufacturers.

The mean corneal astigmatism in this study was 0.96 ± 0.72 D, which is consistent with the values reported from Turkey [26] (1.01 ± 0.8 D) and Germany [29] (0.98 ± 0.78 D). In addition, Duman et al. [25], reported a lower mean corneal astigmatism value in Turkey (0.84 ± 0.70 D), while Sharma et al. [23], reported a slightly higher rate (1.17 ± 1.15 D) in their study in India. The reasons for these differences may include the different characteristics of the populations included in the study and the use of devices with different technical features in obtaining keratometric measurements.

One of the key findings of this study is the nonlinear relationship between age and corneal astigmatism magnitude. While several studies have highlighted the linear relationship between age and the magnitude of corneal astigmatism [30,31], In this study, we showed a strong U-shaped nonlinear relationship between corneal astigmatism and age, with higher astigmatism levels at younger ages, a decrease in the 60–70 age group, and a subsequent increase after the age of 70. A similar relationship was recently demonstrated by Wu et al. [14]. Due to this nonlinear trend, corneal astigmatism correction is difficult in younger patients (<60 years). This finding suggests that corneal astigmatism is a dynamic parameter and does not follow a uniform age-related trajectory across all age groups.

Regarding astigmatism axis, the most common type of astigmatism was WTR astigmatism, followed by ATR astigmatism, which is consistent with reports from European populations [29,32]. However, studies from other regions have reported a predominance of ATR astigmatism [23,33]. Importantly, we found a gradual transition from WTR astigmatism to ATR astigmatism with increasing age. ATR astigmatism increased significantly with age, while WTR astigmatism decreased, findings that are consistent with previous reports [34,35,36]. This age-related shift was further supported by vector analysis, showing a significant decrease in J0 values with age consistent with previous reports [37,38].

From a clinical perspective, the demonstrated association between age and ATR astigmatism has important implications for cataract surgery planning. Our findings suggest that middle-aged patients undergoing cataract surgery may continue to experience a progressive shift toward ATR astigmatism over time. Therefore, corneal astigmatism correction strategies should consider not only the current refractive status but also the expected age-related changes. While this study does not propose a specific correction algorithm, it highlights the importance of individualized surgical planning and cautious interpretation of astigmatism correction targets, particularly in patients with longer life expectancy. Moreover, although postoperative outcomes were not analyzed, the preoperative identification of age-related astigmatic patterns may help surgeons anticipate which patients are more likely to benefit from specific astigmatism-correcting strategies and highlights the need for individualized surgical planning.

Previous studies have shown that corneal astigmatism continues to shift toward ATR even years after cataract surgery [39]. It has been emphasized that this process may be based on several mechanisms including genetics, extraocular muscle tension, visual feedback and eyelid pressure, as well as age-related changes in the corneal structure [39,40,41]. In this context, understanding age-related trends may help surgeons anticipate long-term refractive outcomes and avoid unintended refractive surprises.

The fact that this trend continues after cataract surgery reveals the importance of the type of astigmatism and the method to be preferred in astigmatic correction. Therefore, if astigmatic correction is to be performed, a long-term plan should be made by taking these tendencies into consideration. If corneal astigmatism is fully corrected in middle-aged cataract patients, it should be taken into account that there may be a shift towards ATR astigmatism in later years [42]. Wu et al. [14], noted that ATR astigmatism was positively correlated with age in middle-aged and elderly patients. In this study, there was a significant positive correlation between ATR astigmatism and age in the elderly patient group. Focusing on long-term outcomes in cataract surgery, particularly in the middle-aged group, targeting ATR astigmatism to be fully or overcorrected may prevent worsening of astigmatism in later years [43]. The rate of oblique astigmatism in this study is consistent with the rates reported by several studies [44,45]. In this study, mean J45 did not differ significantly with age, representing a negligible age-related shift in oblique astigmatism, which is consistent with previous reports [17,18,26,38]. These studies, reporting from different geographies and races, indicate smaller variations in oblique astigmatism than in WTR and ATR astigmatism.

This study has some limitations. As a hospital-based, retrospective study, it may not fully represent the general population in Turkey. Also, this study was designed as a preoperative descriptive analysis and therefore did not evaluate postoperative changes in corneal astigmatism or compare different astigmatism-correcting techniques, such as on-axis phacoemulsification, OCCI, LRI, femtosecond laser keratotomy, or toric IOL implantation. In addition, although the study did not include a non-cataract control group, its primary aim was descriptive and epidemiological, focusing on characterizing corneal astigmatism patterns in cataract surgery candidates rather than comparative outcome analysis. Moreover, posterior corneal astigmatism could not be evaluated, as only anterior keratometric data from the IOLMaster 500 were available. It is known that the posterior cornea typically exhibits ATR astigmatism regardless of age [46], which may lead to overcorrection of WTR astigmatism and undercorrection of ATR astigmatism if not considered [47]. Therefore, in order to obtain more effective and long-term results in the correction of corneal astigmatism, posterior corneal astigmatism should also be taken into consideration. Future prospective studies comparing postoperative corneal astigmatism changes among different correction techniques, including on-axis phacoemulsification, OCCI, LRI, femtosecond laser-assisted keratotomy, and toric intraocular lenses, are warranted to validate the clinical implications of these preoperative findings.

## 5. Conclusions

In conclusion, this study represents the largest series from Turkey evaluating the characteristics and age-related changes in corneal astigmatism in cataract surgery candidates. The main findings of the study can be summarized as follows: (1) corneal astigmatism magnitude shows a nonlinear, U-shaped association with age, with higher values in the 40–60 age range, a decrease between 60 and 70 years, and a subsequent increase after the age of 70; (2) the prevalence of ATR astigmatism increases significantly with age, while WTR astigmatism decreases, whereas oblique astigmatism remains relatively stable across age groups; (3) a significant age-related increase in corneal astigmatism is observed specifically in the ATR group, particularly in patients aged 65 years and older. Additionally, more than one-third of patients were eligible for toric IOL implantation. Taken together, these findings underscore the importance of detailed preoperative assessment of both the magnitude and axis of corneal astigmatism. The results may assist surgeons in optimizing astigmatism management strategies and in making informed decisions regarding toric IOL selection in cataract surgery.

## Figures and Tables

**Table 1:** Demographic and clinical characteristics of the study population.

Characteristics	Value
Total number of eyes (*n*)	2152
Age (years)	
Mean ± SD	70.56 ± 8.88
Range	40 to 94
Male, no. (%)	1010 (46.9)
Corneal astigmatism (D)	
Mean ± SD	0.96 ± 0.72
Range	0.00 to 8.31
Mean ± SD (K1)	43.32 ± 1.60
Mean ± SD (K2)	44.28 ± 1.66
J0 (D)	
Mean ± SD	0.05 ± 0.51
Range	−2.29 to 4.38
J45 (D)	
Mean ± SD	0.01 ± 0.30
Range	−1.96 to 1.95
Axis of astigmatism, no. (%)	
With–the-rule	934 (43.4)
Against-the-rule	806 (37.5)
Oblique	412 (19.1)
Axial length (mm)	23.46 ± 1.02
Anterior chamber depth (mm)	3.08 ± 0.40
IOL power (D)	21.04 ± 2.47
Toric IOL, no. (%)	321 (14.9)

Data are presented as numbers and percentages, means and standard deviations. K1: flat keratometry; K2: steep keratometry; J0: Jackson cross-cylinder, axes at 180° and 90°; J45: Jackson cross-cylinder, axes at 45° and 135°; D: diopter; IOL: intraocular lens.

**Table 2:** Spearman’s correlation analysis of corneal astigmatism and age in three types of astigmatism axis.

Corneal Astigmatism	Total		Age < 65 Years		Age ≥ 65 Years	
	*r*	*p* Value	*r*	*p* Value	*r*	*p* Value
With-the-rule astigmatism	−0.056	0.088	−0.217 *	**<0.001**	0.020	0.617
Against-the-rule astigmatism	0.148 *	**<0.001**	−0.098	0.311	0.153 *	**<0.001**
Oblique astigmatism	0.098	0.078	0.047	0.653	0.077	0.171

Significant *p* values are shown in bold. * Spearman’s correlation test.

## Data Availability

The raw data supporting the conclusions of this article will be made available by the authors on request.
